# Primary health care in China

**DOI:** 10.1016/j.lanwpc.2020.100019

**Published:** 2020-10-28

According to the latest Global Burden of Diseases, Injuries, and Risk Factors Study (GBD) 2019, published in *The Lancet* on Oct 17, 2020, the mean life expectancy at birth in 2019 for China is 80-8 years for women and 74-7 years for men, and the under-5 mortality is 8-5 deaths per 1000. This improvement in life expectancy and mortality is remarkable, partly owing to health-care reform in China in the past decade, in which one of the aims was to increase equal access to health care and financial protection for Chinese citizens, especially the most susceptible ones.

Despite advances being made, China still faces many health challenges. As indicated in GBD 2019, stroke, ischaemic heart disease, and chronic obstructive pulmonary disease along with other non-communicable diseases are the major causes of death in China. The rising rate of non-communicable diseases is fuelled by an ageing population, urbanisation, and unhealthy lifestyles, such as tobacco use and dietary risks. Relying on a hospital-centric health-care system cannot meet the growing needs of the population. As the anvil of an effective health-care system, the shift to primary health care, including village clinics, town health centres, and urban community health centres, can help to relieve the pressure on health-care systems by providing clinical care and basic public health services at the local level.

Although primary health care has proven to be an efficient and cost-effective way to provide health-care services, its use in China is lower than expected. According to a review by Shengshou Hu and colleagues, published June 6, 2020, in *The Lancet*, insufficient education and training for primary health-care workers, over-testing and over-treatment due to the fee-for-service payment system, fragmented health care from hospital and primary health care, and poor continuity of care are the major challenges of providing a better primary health-care service in China. Patients’ concerns about the suboptimal quality of the health service, and the competition from hospitals due to the fee-for service payment system make it difficult for primary health care to attract and retain patients.

In two research Articles published in *The Lancet Regional Health -- Western Pacific*, Hongmei Yi and colleagues provided support for the view that village clinicians from rural areas in China were insufficiently trained to diagnose, treat, and manage chronic diseases to meet patient need. In one paper, the village clinicians carried out only 39% of questions and examinations essential for a correct diagnosis, and only 20% of them made a correct diagnosis for unstable angina. The other study showed a better diagnosis performance: 63% of village clinicians made a correct diagnosis for childhood epilepsy, but on average, only 14% of essential questions and health checks were completed. To improve the quality of primary health care, authors recommended enhancing the training quality for primary health-care workers. It is a dilemma. Primary health workers were expected to take on more duties for chronic disease diagnosis and management, but as pointed out by Benjamin Daniels in the linked commentary for the two papers, “what primary care providers have revealed themselves to be supremely competent at is not diagnosis and treatment, but risk assessment and triage”.

The two research studies and the commentary from our journal show the complexity of understanding primary health care and the barriers to its improvement. In 2016, China announced a long-term health-care vision called *Healthy China* 2030. It reflects the Chinese Government’s willingness to improve health services and prioritise health in all policies. Although China has gradually recovered from the COVID-19 pandemic, moving away from hospital-based care is especially important for the nation to cope with current and future health challenges. To develop a strong primary health-care system that can provide high-quality and continuous care to all citizens equally will be a crucial step towards achieving the long-term health-care goals set out in *Healthy China* 2030.

*The Lancet Regional Health – Western Pacific*

